# Behavioral Modeling of Memristors under Harmonic Excitation

**DOI:** 10.3390/mi15010051

**Published:** 2023-12-26

**Authors:** Elena Solovyeva, Artyom Serdyuk

**Affiliations:** Department of Electrical Engineering Theory, Saint Petersburg Electrotechnical University “LETI”, 197022 St. Petersburg, Russia; serduk_artem_99@mail.ru

**Keywords:** behavioral modeling, nonlinear model, split signals, neural network, memristor, memristor model

## Abstract

Memristors are devices built on the basis of fourth passive electrical elements in nanosystems. Because of the multitude of technologies used for memristor implementation, it is not always possible to obtain analytical models of memristors. This difficulty can be overcome using behavioral modeling, which is when mathematical models are constructed according to the input–output relationships on the input and output signals. For memristor modeling, piecewise neural and polynomial models with split signals are proposed. At harmonic input signals of memristors, this study suggests that split signals should be formed using a delay line. This method produces the minimum number of split signals and, as a result, simplifies behavioral models. Simplicity helps reduce the dimension of the nonlinear approximation problem solved in behavioral modeling. Based on the proposed method, the piecewise neural and polynomial models with harmonic input signals were constructed to approximate the transfer characteristic of the memristor, in which the current dynamics are described using the Bernoulli differential equation. It is shown that the piecewise neural model based on the feedforward network ensures higher modeling accuracy at almost the same complexity as the piecewise polynomial model.

## 1. Introduction

Technological progress is closely related to the emergence of new materials, the improvement and development of technologies and equipment, and their active implementation into production. One example of this progress is the creation of technical devices and systems based on memristors [1,2,3,4,5,6,7,8].

A memristor is a resistor with memory, known as the fourth passive electrical element (other elements: resistor, capacitor, inductance), which was theoretically described by L. Chua in 1971 [9]. The resistance of a memristor nonlinearly depends on the history of changes in its current, i.e., the current at a given moment in time and a past moment in time.

The intensive use of the memristor began with its physical creation based on the nanoscale metal-dielectric-metal structure at Hewlett Packard in 2008 [10]. The physical memristor consisted of two platinum nanowire electrodes separated by a semiconductor material (approximately 5 nm of titanium dioxide film). On one side of the film, a doping impurity was added, and an oxygen-depleted TiO_2−x_ layer was formed, in which an oxygen deficiency was observed in the form of positive vacancies. When the contact voltage was positive, positive oxygen vacancies repelled and moved into the insulating (undoped) layer of titanium dioxide TiO_2_. As a result, the boundary between the layers moved the width of the conducting TiO_2−x_ layer, and the memristor conductivity increased. The more conductive state of the memristor is called “set or reset”. With negative voltage polarity across the contact mentioned, the attraction of positive oxygen vacancies, a decrease in the width of the conducting TiO_2−x_ layer, and an increase in the memristor resistance were observed. The less conductive state of the memristor is called “off or erasing”. After switching off the voltage, the memristor did not change its state, i.e., it “remembered” the last value of conductivity (resistance).

The developed technologies of memristors underlie the following classification [1,2,3,4,5,6,7,8]:

- Resistive memristors with metal-dielectric-metal structures (various oxides are used as a dielectric, for example, titanium, gadolinium, aluminum, hafnium, graphene). Memristor activity is associated with oxidation–reduction (redox) reactions;
- Spintronic magnetic memristors, the resistances of which vary in accordance with their magnetization (in accordance with electron spins). Here, the electron is represented as a spinning ball and possesses a quantum mechanical property called “spin”. Spin characterizes magnetism; for instance, materials are magnetized when most electrons have codirectional spins;
- Memristors with transition from one phase state (polycrystalline) to another (amorphous) and vice versa;
- Ferroelectric memristors based on ferroelectric tunnel connections. The memristor consists of two metal electrodes with a thin ferroelectric layer (material changing electrical polarization spontaneously) in between. Polarization switching under the influence of an external electric field changes the memristor resistance;
- Organic (polymer) memristors, in which electrochemical reactions are a mechanism for switching resistance.

Different memristors possess the following general properties [1,2,3,4,5,6]:

- Memristor resistance in analog mode can take any value, not only 0 or 1. Such variability can be implemented on one element, wherein the memristor size is reduced to several nanometers, and the response speed is reduced to nanoseconds;
- The memristor does not store its properties in the form of a charge. Thus, it is resistant to charge leakages, which have to be dealt with when switching to nanometer-scale microcircuits. It is completely nonvolatile, and data can be stored as long as the materials used for its implementation exist;
- Memristors placed on crossbars can be used to form densely packed memory;
- Low power consumption by the memristor makes it a very promising element in energy-efficient areas. For instance, contemporary high-performance computing and cooling systems consume tens of megawatts of energy. The systems based on memristors open the way to energy saving in this area;
- A lot of memristor materials are compatible with CMOS technology.

Memristors have a wide field of application: electrical and radio engineering, telecommunication and control systems, computing and neuromorphic systems, robotics, cryptography, etc. Memristors are the basis of multifunctional devices, such as, in the analog field:

- Oscillators, variable gain amplifiers, nonlinear filters, Schmidt trigger, and rectifiers [1,2,3,4,5,6,7,8];
- Chains with chaotic vibrations [11,12,13,14];
- Neural networks (direct propagation, recurrent, cellular) performing modeling, filtering, signal generation, object management, and speech and image processing [3,13,15,16,17,18,19,20,21];
- Neuromorphic systems that imitate the functioning of nerve tissues or biological brain structures [3,6,8,22,23,24];
- Programmable analog integrated circuits [3,8,25].

In the digital field:

- Logic circuits, reconfigurable logic circuits, digital gates, and associative memories (content-addressable memory) [8,25,26];
- Nonvolatile storage devices [27].

Models of memristive devices are presented in analytical form (systems of nonlinear differential equations), as well as numerically in software environments [1,2,4,28]. Memristor models are based on physical mechanisms and their synthesis technologies [3,6,29,30,31,32,33].

Models of memristors, which are implemented on metal oxides, are widespread. For instance, the linear ionic drift model (the HP model) [10], nonlinear ionic drift models with different weight functions (Benderli model, Joglekar model, Biolek model, Prodromakis model, Zha model, Yakopcic model, Ascoli–Corinto model), nonlinear Lehtonen-Laiho model, Simmons tunnel barrier model with nonlinear and asymmetric switching characteristics), and the threshold adaptive memristor model (the TEAM model) [28,32,33]. The variety of physical principles underlying the technologies for creating memristors and the wide range of applications of memristor-based devices set the task to develop mathematical memristor models that are universal in relation to the physics of modeled processes and the types of input and output signals of devices, which are different in the application fields.

The “black box” (behavioral) models are universal. Within the framework of the “black box” principle, behavioral modeling of the object, which is presented as a “black or gray box”, establishes the unique input–output correspondence between the sets of the input and output signals [34,35,36,37]. Universal behavioral models include the following:

- The functional Volterra series in time and frequency forms [37,38,39,40,41];
- Regression models, mostly recurrent [37];
- Models based on split signals [42,43].

The functional Volterra series has a severe handicap. It is used in its convergence region, so it is applicable in a weakly nonlinear mode of operation. Regression models can be used in a severely nonlinear mode. However, regression models tend to have feedback, which results in the problem of ensuring the stability of nonlinear processes. While the methods of ensuring stability in recurrent linear systems are known, there are no similar general methods for recurrent nonlinear systems.

Modeling based on split signals is advisable. Splitting means that the input scalar signal is converted into a vector signal so that the phase portrait does not intersect, touch, or cross zero. Splitting is necessary to ensure the unique input–output correspondence. The elements of the split signal vector are used as basic functions in nonlinear models, such as multidimensional polynomials, fractional functions, and neural networks. The model parameters result from solving the approximation task in the mean square norm. Thus, the dynamics and dimensionality of the model are formed using signal splitting, and the model nonlinearity is formed using the nonlinear inertialess transformation of split signals [35,42,43]. The splitting operation is performed for the input signal, so the behavioral model is adapted to the input signals. This adaptation makes the model simpler in comparison with a universal model, such as the Volterra series.

The paper contains four sections. The importance of memristors in many areas of technique, the variety of technologies, and consequently, models for memristors, and the building of universal behavioral models, especially models based on split signals, which have a number of advantages in comparison with other behavioral models, have been described in the Introduction. Behavioral modeling with split signals and splitting harmonic input signals using a delay line are presented in Section 2. The modeling results of the memristors, in which the dynamics of currents are described using the Bernoulli differential equation, the used piecewise polynomial and neural models, their comparison, and the corresponding conclusions are presented in Section 3 and Section 4.

## 2. Behavioral Model with Split Signals

**Figure 1:**
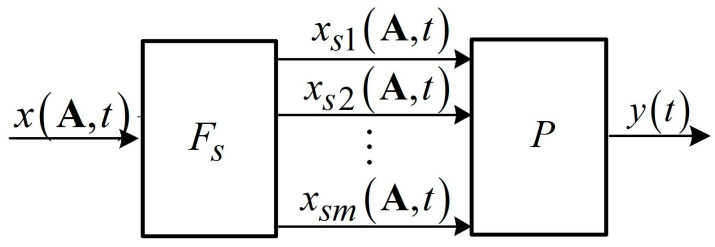
The block scheme of a model with split signals.

According to the splitting method [35,42,43], the operator of a nonlinear device $F_{\varepsilon }$ is composed of two operators: the splitter operator $F_{s}$ and the operator of a nonlinear inertialess converter P . The block scheme of a model with split signals is shown in Figure 1.

The splitter $F_{s}$ converts the scalar input signals x(A,t)∈X of the device, where $A\in G_{a}$ is the vector of signal parameters from the set $G_{a}$ , into the following vector signals:

(1) $$X_{s}\left(A,t\right)=F_{s}\left[x\left(A,t\right)\right]={\left[x_{s1}\left(A,t\right),\;x_{s2}\left(A,t\right),\;\ldots ,\;x_{sm}\left(A,t\right)\right]}^{T},$$

where T is the transposition sign. The vector signals (1) are split on the set of their existence. This means that they obey the following conditions:

- The vector signals are not zero;
(2) $$X_{s}\left(A,t\right)\neq 0,$$
  at all $A\in G_{a}$ , $t\in G_{t}$ ;
- The vector signals are different at every point in time, i.e., at any, $A_{\alpha }\neq A_{\beta }$ , $A_{\alpha }\in G_{a}$ , $\;A_{\beta }\in G_{a}$ , and $t_{\alpha }\neq t_{\beta }$ , $t_{\alpha }\in G_{t}$ , $t_{\beta }\in G_{t}$ , inequality
(3) $$X_{s}\left(A_{\alpha },t_{\alpha }\right)\neq X_{s}\left(A_{\beta },t_{\beta }\right),$$
  is true.

As the analysis of inequalities (2) and (3) shows, splitting means that the phase portrait based on $X_{s}\left(A,t\right)$ does not intersect, touch, or cross zero [11,30,31,44].

The signal splitting is realized using linear and nonlinear and stationary and nonstationary converters [35,36,42]. Splitting depends on the class of the input scalar signal. An important criterion for the selection of a splitter is the minimum number of the splitter channels (minimum length of the split signal vector). The smaller the length of the vector $X_{s}\left(A,t\right)$ , the smaller the dimensionality of the model. As a result, the model is simplified.

**Figure 2:**
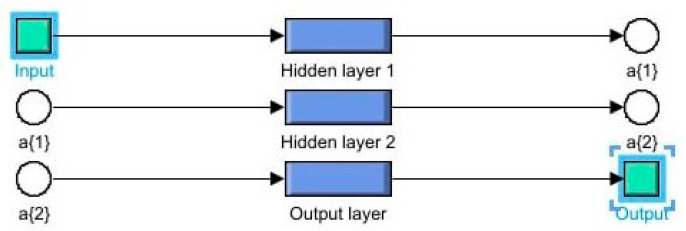
The convertor structure in the form of a three-layer feedforward neural network.

The operator P of a nonlinear inertialess converter (Figure 1) maps the vector signals $X_{s}\left(A,t\right)$ into the scalar output signals y(t)∈Y of the model. The mathematical forms of the converter are different, for instance, polynomials, fractions, neural networks. The converter schemes are both non-recursive and recursive [35,36,42]. For example, the polynomial is of the form:

(4) $$P\left[X_{s}\left(A,t\right)\right]=\sum_{j_{1}=0}^{J_{1}}\;\sum_{j_{2}=0}^{J_{2}}\;\ldots \sum_{j_{m}=0}^{J_{m}}C_{j_{1}j_{2}\;\ldots \;j_{m}}{\left[x_{s1}\left(A,t\right)\right]}^{\;j_{1}}{\left[x_{s2}\left(A,t\right)\right]}^{\;j_{2}}\ldots \;\;\ldots \;{\left[x_{sm}\left(A,t\right)\right]}^{\;j_{m}},$$

the power of polynomial (4) is equal to $J=\sum_{r=1}^{m}\;J_{r}$ . The structure of the three-layer feedforward neural network is shown in Figure 2.

In Figure 2, the block “Input” contains a vector of split signals. These signals are processed in two consecutive hidden layers of “Hidden 1” and “Hidden 2”, which comprise several neurons with nonlinear activation functions. Then, the layer “Hidden 2” is followed by “Output layer” with a neuron and the linear activation function. As a result, a scalar signal is formed at the output of the neural network. Behavioral modeling based on the three-layer network provides a higher modeling accuracy than the two-layer network. This fact is justified by the fact that the first hidden layer describes the local properties of processes by dividing the operator space into subspaces and the second hidden layer describes the global properties of processes by combining the output signals of the neurons of the first hidden layer [45].

Harmonic signals are the input signals of memristors and memristive devices to build transfer characteristics. In addition, harmonic signals are an important class for analyzing dynamic devices, namely, to determine the frequency characteristics, calculate the steady-state processes, and perform spectral analysis.

Therefore, the common task is to construct a splitter for harmonic signals. Below, we describe the solution of this task and present the resulting splitter structure as a delay line.

### 2.1. Splitting of Harmonic Signals with Amplitude Equal One

Let us consider a class of sine wave signals with an amplitude equal to one:

(5) $$x(t)=\sin(\omega_{0}t)=\sin(\bar{t}),$$

where $\omega_{0}=2\pi /T_{x}$ is the angular frequency; $T_{x}$ is the signal period; and $\bar{t}=\omega_{0}t$ , $\bar{t}\in \left[0,\;2\pi \right)$ is the normalized continuous time.

**Statement 1.**  *The split signal on set* $\bar{t}\in \left[0,\;2\pi \right)$ *is a vector*

(6) $$X_{s}(\bar{t})={\left[\sin(\bar{t}),\;\sin(\bar{t}-{\bar{t}}_{0})\right]}^{T},$$

*where* ${\bar{t}}_{0}$ *is a constant, denoting a shift in normalized time, and* T *is the transposition sign.*

**Figure 3:**
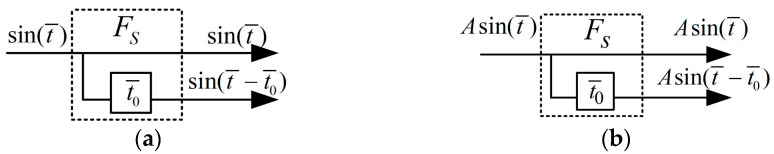
The block scheme of the splitter as a delay line for harmonic signal with an amplitude equal to one (**a**) and variable (**b**).

The block scheme of the splitter with the delay element based on Statement 1 is depicted in Figure 3a. Now we can prove Statement 1.

**Proof of Statement 1.**  To prove the splitting of signal (6), we consider the following equalities:

(7) $$\sin({\bar{t}}_{\alpha })=\sin({\bar{t}}_{\beta }),$$

(8) $$\sin({\bar{t}}_{\alpha }-{\bar{t}}_{0})=\sin({\bar{t}}_{\beta }-{\bar{t}}_{0}).$$

On the basis of trigonometric formulas, we convert the equality (8) to the form:

$$\sin({\bar{t}}_{\alpha })\cos({\bar{t}}_{0})-\cos({\bar{t}}_{\alpha })\sin({\bar{t}}_{0})=\sin({\bar{t}}_{\beta })\cos({\bar{t}}_{0})-\cos({\bar{t}}_{\beta })\sin({\bar{t}}_{0}),$$

in view of equality (*7*), we write:

$$\sin({\bar{t}}_{\alpha })\cos({\bar{t}}_{0})-\cos({\bar{t}}_{\alpha })\sin({\bar{t}}_{0})=\sin({\bar{t}}_{\alpha })\cos({\bar{t}}_{0})-\cos({\bar{t}}_{\beta })\sin({\bar{t}}_{0}),$$

from this, we get:

$$\cos({\bar{t}}_{\alpha })=\cos({\bar{t}}_{\beta }),$$

or

(9) $$\sin(\pi /2+{\bar{t}}_{\alpha })=\sin(\pi /2+{\bar{t}}_{\beta }).$$

Thus, instead of Equations (7) and (8), we analyze Equations (7) and (9).

**Figure 4:**
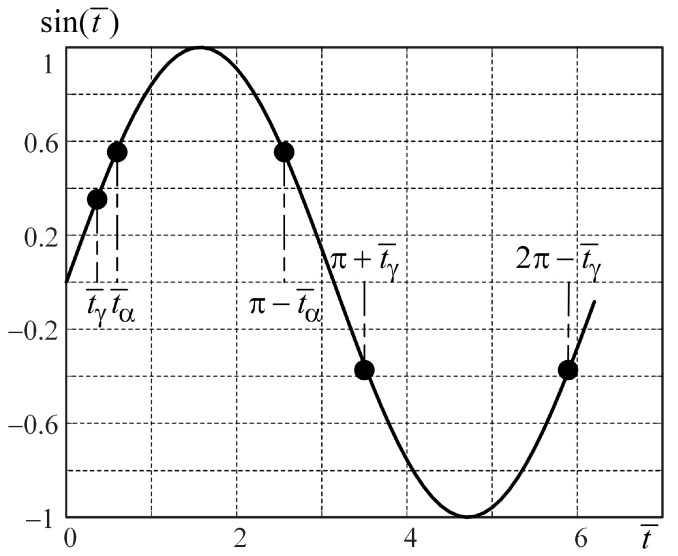
Signal with non-splitting values.

Figure 4 shows the signal $\sin(\bar{t})$ . As one can see in Figure 4, two areas (with positive and negative signal values) should be considered.

On the time interval $\bar{t}\in \left[0,\;\pi \right]$ , equality (7) is true under two conditions:

(10) $${\bar{t}}_{\beta }={\bar{t}}_{\alpha },$$

(11) $${\bar{t}}_{\beta }=\pi -{\bar{t}}_{\alpha },$$

where ${\bar{t}}_{\alpha }\in \left[0,\;\pi \right]$ , ${\bar{t}}_{\beta }\in \left[0,\;\pi \right]$ .

Equality (9) is true under condition (10) and not true under condition (11), as

$$\sin(\pi /2+{\bar{t}}_{\alpha })\neq \sin(\pi /2+(\pi -{\bar{t}}_{\alpha })),$$

$$\sin(\pi /2+{\bar{t}}_{\alpha })\neq \sin(-\pi /2-{\bar{t}}_{\alpha }),$$

$$\sin(\pi /2+{\bar{t}}_{\alpha })\neq -\sin(\pi /2+{\bar{t}}_{\alpha }).$$

On the time interval $\bar{t}\in \left[\pi ,\;2\pi \right)$ , equality (7) is true under two conditions: one of which is expression (10), the other is the following:

(12) $${\bar{t}}_{\alpha }=\pi +{\bar{t}}_{\gamma },\;{\bar{t}}_{\beta }=2\pi -{\bar{t}}_{\gamma },$$

where ${\bar{t}}_{\alpha }\in \left[\pi ,\;2\pi \right)$ , ${\bar{t}}_{\beta }\in \left[\pi ,\;2\pi \right)$ , ${\bar{t}}_{\gamma }\in \left[0,\;\pi \right)$ .

Equality (9) is true under condition (10) and not true under condition (12), as

$$\sin(\pi /2+(\pi +{\bar{t}}_{\gamma }))\neq \sin(\pi /2+(2\pi -{\bar{t}}_{\gamma })),$$

$$\sin(-\pi /2+{\bar{t}}_{\gamma })\neq \sin(\pi /2-{\bar{t}}_{\gamma }),$$

$$-\sin(\pi /2-{\bar{t}}_{\gamma })\neq \sin(\pi /2-{\bar{t}}_{\gamma }).$$

Thus, Equations (7) and (9), as well as (7) and (8) are true only under condition (10). Note that on the time interval $\bar{t}\in \left[0,\;2\pi \right)$ , the vector $X_{s}(\bar{t})$ from expression (6) does not equal zero.

Eventually, signals in vectors ${\left[\sin(\bar{t}),\;\sin(\pi /2+\bar{t})\right]}^{T}$ and ${\left[\sin(\bar{t}),\;\cos(\bar{t})\right]}^{T}$ , as well as in vector $X_{s}(\bar{t})$ from Formula (6) are split on a set. □

### 2.2. Splitting of Harmonic Signals with Variable Amplitude

Let us consider a class of sine wave signals with variable amplitude:

(13) $$x(A,\bar{t})=A\sin(\bar{t}),$$

where A>0 , $A\in G_{A}$ , $G_{A}$ is the value set of variable A , $\bar{t}\in \left[0,\;2\pi \right)$ .

**Statement 2.**  *The signal split with respect to variable* A *and* $\bar{t}$ *on set* $G_{A}\times \left[0,\;2\pi \right)$ *is a vector*

(14) $$X_{s}(A,\bar{t})={\left[A\sin(\bar{t}),\;A\sin(\bar{t}-{\bar{t}}_{0})\right]}^{T},$$

*where* ${\bar{t}}_{0}$ *is a constant representing the time shift,* T *is the transposition sign.*

The block scheme of the splitter with the delay element based on Statement 2 is shown in Figure 3b. Now we can prove Statement 2.

**Proof of Statement 2.**  From the vector signal (14), we come to a vector signal:

(15) $${\left[A\sin(\bar{t}),\;A\cos(\bar{t})\right]}^{T},$$

and analyze the splitting of its elements.

For this, we form a pair of equations:

(16) $$A_{\alpha }\sin({\bar{t}}_{\alpha })=A_{\beta }\sin({\bar{t}}_{\beta }),$$

(17) $$A_{\alpha }\cos({\bar{t}}_{\alpha })=A_{\beta }\cos({\bar{t}}_{\beta }).$$

Then, we determine the squares of Equations (16) and (17), add them together, and obtain:

(18) $$A_{\alpha }^{2}=A_{\beta }^{2}.$$

Equality (18) is true under conditions: $A_{\beta }=A_{\alpha }$ and $A_{\beta }=-A_{\alpha }$ . Because A>0 , we keep only the following expression:

(19) $$A_{\beta }=A_{\alpha }.$$

Instead of Equations (16) and (17), it is sufficient to analyze Equations (7) and (9), taking into account equality (19). On proving the splitting vector (6), equalities (7) and (9) are shown to be true under condition (10). It should be emphasized that vectors (14) and (15) are not zero on the interval $\bar{t}\in \left[0,\;2\pi \right)$ .

Thus, vector (15) and, consequently, vector (14) are split on the set $G_{A}\times \left[0,\;2\pi \right)$ . □

The vectors (6) and (14) of split signals constructed for signal classes (5) and (13), respectively, are basic functions of two-dimensional polynomial (4) ( m=2 ).

As a consequence, the splitter in the form of a delay line is synthesized for the widely used class of harmonic signals. This splitter ensures the minimum possible memory length of nonlinear models. The implementation of the delay line is convenient in digital technology developed for radio and telecommunication systems, control systems, neurocomputers, robotic complexes, etc.

## 3. Behavioral Modeling of the Bernoulli Memristors

As an example of modeling with the help of multidimensional polynomials and neural networks based on split signals, we consider the behavioral modeling of memristors, in which the dynamics of currents are described using the Bernoulli differential equation. These memristors are called “Bernoulli memristors”. The Bernoulli memristors belong to the class of ideal memristors [44,46]. The element controlled by charge and excited by voltage is described using a pair of equations [44]:

$$\frac{dq(t)}{dt}=i(t),$$

$$i(t)=\frac{1}{M\left(q(t)\right)}v(t),$$

where *q*(*t*) is a charge (control signal or memristor state variable); *v*(*t*), *i*(*t*) are the voltage (input) and current (output) signals, respectively; and *M*(*q*(*t*)) is a memristance.

The current dynamics are determined using the Bernoulli differential equation [44,46]:

(20) $$\frac{di(t)}{dt}-\frac{dv(t)/dt}{v(t)}i(t)=-\frac{k_{2}}{v(t)}i^{\alpha +2}(t),$$

where α≠−1,−2 is an integer and $k_{2}=\frac{dM\left(q(t)\right)}{dq(t)}$ is a variable related to the physical structure of the memristor.

The memristance dynamics are described using expression [44,46]:

$$\frac{dM\left(q(t)\right)}{dt}=k_{2}i^{\alpha }(t)=\frac{dM\left(q(t)\right)}{dq(t)}i^{\alpha }(t).$$

The behavioral models, which we construct, approximate the transfer characteristics (functional operators) to establish correspondence between the set of the input and output signals. The input signal of the Bernoulli memristor is the voltage applied to the memristor, and the output signal is the current flowing through it. Thus, the transfer characteristic is the dependence of current on voltage i(v) .

The set of the input signals of the Bernoulli memristor include harmonic signals described as:

(21) $$v(\bar{t})=A\sin(\omega_{0}t)=x(A,\bar{t})=A\sin(\bar{t}),$$

where *A*, A∈(−0.5;1] is an amplitude; $\omega_{0}$ is the angular frequency; and $\bar{t}=\omega_{0}t$ , $\bar{t}\in \left[0,\;2\pi \right)$ is the normalized continuous time.

The set of output signals is developed using the equality [36,44]:

(22) $$i(\bar{t})=\frac{v(\bar{t})}{M_{0}{\left[1+\beta_{2}\int_{0}^{\bar{t}}v^{\alpha }(\tau )d\tau \right]}^{1/(\alpha +1)}},$$

where $M_{0}=v(0)/i(0)$ , $\beta_{2}=(\alpha +1)k_{2}A/M_{0}^{\alpha +1}$ . Expression (22) is the analytical solution of Equation (20) in view of the input signal (21) [44].

When assigning variable α , for instance, α=1 and input (21), we modify equality (22) and obtain [44]:

(23) $$i(\bar{t})=\frac{v(\bar{t})}{M_{0}{\left[1+\beta_{2}\left(1-\cos(\bar{t})\right)\right]}^{1/2}}.$$

Furthermore, when $M_{0}=2$ , $k_{2}=2$ , we get $\beta_{2}=A$ , and from expression (23), we come to the formula:

(24) $$i(\bar{t})=\frac{v(\bar{t})}{2{\left[1+A\left(1-\cos(\bar{t})\right)\right]}^{1/2}}.$$

Thus, the set of the memristor output signals includes the current signals $i(\bar{t})$ , obtained from expression (24) at the input voltage signals $v(\bar{t})$ from (21).

As follows from the analysis of expressions (22)–(24), the nonlinearity of the transfer characteristic depends severely on the amplitude of the input signal. This is why it is advisable to build piecewise behavioral models on subsets of the input and corresponding output signals. Hence, the general approximation task of high dimension is divided into several subtasks of low dimension. These subtasks are solved using the test input signals of different amplitudes from the subsets of the impact.

For Bernoulli memristor modeling, the amplitude range of the input signal is divided into five subranges: (−0.5; −0.45], (−0.45; −0.4], (−0.4; 0), (0; 0.5), [0.5; 1]. Within each subrange, we chose some amplitudes to build (train) models. These amplitudes form the following vectors:

$$\begin{array}{l} A_{1}=\left[–0.48;\;–0.47;\;–0.46;\;–0.45\right],\\ A_{2}=\left[–0.449;\;–0.44;\;–0.43;\;–0.42;\;–0.41;\;–0.4\right],\\ A_{3}=\left[–0.399;\;–0.3;\;–0.2;\;–0.1;\;–0.001\right],\\ A_{4}=\left[0.001;\;0.1;\;0.2;\;0.3;\;0.4;\;0.499\right],\\ A_{5}=\left[0.5;\;0.6;\;0.7;\;0.8;\;0.9;\;1\right], \end{array}$$

where 1, 2, …, 5 are the indices of vectors, which denote the numbers of the subranges mentioned above.

**Figure 5:**
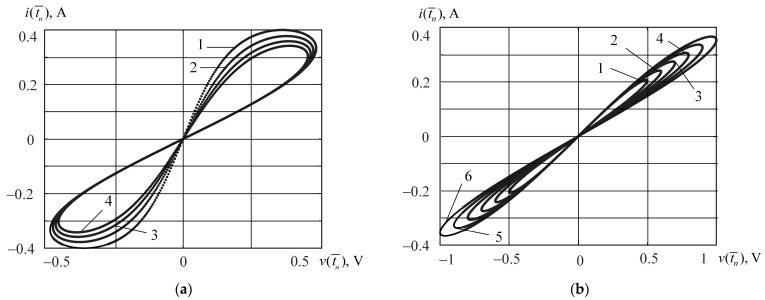
The pinched hysteresis current-voltage relationships corresponding to the subranges (−0.5; −0.45] (**a**) and [0.5; 1] (**b**) of the input signal amplitude.

Figure 5 shows the pinched hysteresis current-voltage relationships of the Bernoulli memristor. The transfer characteristics corresponding to the subranges (−0.5; −0.45] and [0.5; 1] of the input signal amplitude are depicted in Figure 5a,b. The curve numbers correspond to the numbers of an elements in vectors **A**_1_ (Figure 5a) and **A**_5_ (Figure 5b). Input signals (21) with amplitudes from vectors **A**_1_ and **A**_5_, as well as the output signals calculated using formula (24) were used to define the represented transfer characteristics.

From Figure 5a,b, we see the significant dependence of the transfer characteristic on the input signal amplitude. This fact confirms the above-mentioned expediency of building piecewise behavioral models on the subsets of the input signal.

Behavioral models describing the transfer characteristics of the Bernoulli memristors are constructed in piecewise polynomial and neural forms. The splitter is described using the expression (14) and has the structure shown in Figure 3b.

The piecewise polynomial model of the memristor contains five polynomials constructed on the subsets of the input harmonic signal, taking into account the signal amplitudes from vectors **A**_1_, **A**_2_, …, **A**_5_. For each subset, a polynomial is formed on the basis of expression (4) at m = 2 (the number of splitting channels) as the following:

(25) $$y_{k}(\bar{t})=\sum_{j_{1}=0}^{J_{1}}\sum_{j_{2}=0}^{J_{2}}C_{k,j_{1}j_{2}}{\left[x_{s1}\left(A,\bar{t}\right)\right]}^{\;j_{1}}{\left[x_{s2}\left(A,\bar{t}\right)\right]}^{\;j_{2}}=\;=\sum_{j_{1}=0}^{J_{1}}\sum_{j_{2}=0}^{J_{2}}C_{k,j_{1}j_{2}}{\left[A\sin(\bar{t})\right]}^{\;j_{1}}{\left[A\sin(\bar{t}-{\bar{t}}_{0})\right]}^{\;j_{2}},$$

where $x_{s1}(A,\bar{t}),\;x_{s2}(A,\bar{t})$ are the basis functions of the multidimensional polynomial (the elements of the split signals vector (14)), *k* is the number of the input signal subset (of the subrange of the input signal amplitude), and $J=\sum_{r=1}^{2}J_{r}$ is the power of the polynomial. At the first subset of the input signal, the power of polynomial (25) is *J* = 6 (the number of the polynomial parameters is 27), and at other subsets, the power is *J* = 5 (the parameters number of every polynomial is 20). The total number of parameters of the piecewise polynomial model is 107.

The piecewise neural model of the memristor contains five three-layer neural networks. Every neural network has the structure shown in Figure 2. The number of neurons in the hidden layers is chosen so that its increase minimally reduces the modeling error. Thus, the size of every three-layer neural network is 3 × 2 × 1. The activation functions in the hidden layers are specified as hyperbolic tangents. The total number of the parameters of the piecewise neural model is 100. Neural networks are trained in the MATLAB system using the back propagation method with the Levenberg-Marquardt optimization algorithm, which is the combination of the gradient and Newton-Gauss methods and has a high convergence rate [45].

Three test input signals ( θ = 1, 2, 3) different from trained signals are assigned within every subset of the input signal to evaluate the accuracy of piecewise polynomial and neural models. The amplitudes of these test signals in five subsets form the following vectors:

$$\begin{array}{l} A_{c1}=\left[–0.475;\;–0.465;\;–0.455\right],\\ A_{c2}=\left[–0.445;\;–0.425;\;–0.405\right],\\ A_{c3}=\left[–0.35;\;–0.25;\;–0.05\right],\\ A_{c4}=\left[0.05;\;0.25;\;0.45\right],\\ A_{c5}=\left[0.55;\;0.75;\;0.95\right], \end{array}$$

where the numeric index (1, 2, …, 5) of a vector denotes the subset number. It should be emphasized that the elements of vectors **A**_c1_, **A**_c2_, …, **A**_c5_ of the amplitudes of the input test signals do not match the elements of vectors **A**_1_, **A**_2_, …, **A**_5_ of the amplitudes of input signals used for training models.

The modeling errors are the following:

The absolute error

(26) $$\Delta_{k,\theta }({\bar{t}}_{n})=i({\bar{t}}_{n})-y_{k,\theta }({\bar{t}}_{n}),n=1,\;2,\;\ldots ,\;628,\;k=1,\;2,\ldots ,\;5,\;\theta =1,\;2,\;3,$$

and the normalized (reduced) absolute error

(27) $${\bar{\Delta }}_{k,\theta }({\bar{t}}_{n})=\left(i({\bar{t}}_{n})-y_{k,\theta }({\bar{t}}_{n})\right)/\underset{{\bar{t}}_{n}\in \left(0,\;2\pi \right)}{\max}\left(\left|i({\bar{t}}_{n})\right|\right),n=1,\;2,\;\ldots ,\;628,\;k=1,\;2,\ldots ,\;5,\;\;\theta =1,\;2,\;3,$$

where ${\bar{t}}_{n}$ is the value of the normalized time varying in the interval $y_{1}({\bar{t}}_{n})$ with the step of 0.01; $i({\bar{t}}_{n})$ is the output signal determined from Equation (24); $y_{k,\theta }({\bar{t}}_{n})$ is the output signal of a piecewise model; k is the number of the input signal amplitude range; θ is the input test signal number;

The maximum error is:

(28) $${\bar{\Delta }}_{\max}(k)=\underset{\theta =1,\;2,\;3}{\max}\left(\underset{{\bar{t}}_{n}\in \left(0,\;2\pi \right)}{\max}\left(\left|{\bar{\Delta }}_{k,\theta }({\bar{t}}_{n})\right|\right)\right)n=1,\;2,\;\ldots ,\;628,\;k=1,\;2,\ldots ,\;5,\;\;\theta =1,\;2,\;3;$$

and the root mean square error is:

(29) $$\varepsilon_{k,\theta }=\sqrt{\frac{1}{Q}\sum_{q=1}^{Q}{\left(\;\frac{i({\bar{t}}_{q})-y_{k,\theta }({\bar{t}}_{q})}{\underset{{\bar{t}}_{n}\in \left(0,\;2\pi \right)}{\max}\left(\left|i({\bar{t}}_{n})\right|\right)}\;\right)}^{\;2}},Q=628,k=1,\;2,\ldots ,\;5,\;\theta =1,\;2,\;3,\;\varepsilon (k)=(\varepsilon_{k,1}+\varepsilon_{k,2}+\varepsilon_{k,3})/3.$$

**Figure 6:**
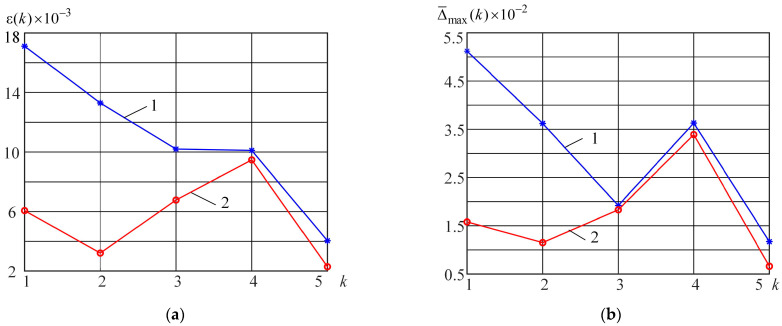
Error $\varepsilon_{k}$ (**a**) and ${\bar{\Delta }}_{\max}(k)$ (**b**) obtained using the piecewise polynomial and neural models.

The root mean square (Equation (29)) and maximum (Equation (28)) errors of modeling are shown in Figure 6a,b respectively. Curve 1 (blue) is obtained for the piecewise polynomial model, and Curve 2 (red) is obtained for the piecewise neural model.

**Figure 7:**
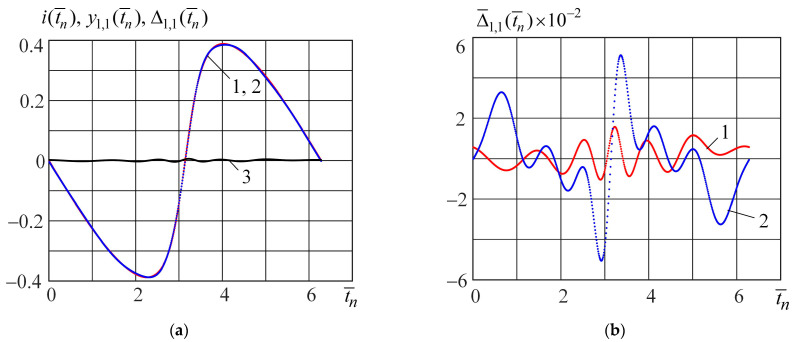
The output signals $i({\bar{t}}_{n}),\;y_{1,1}({\bar{t}}_{n}),\;\Delta_{1,1}({\bar{t}}_{n})$ (**a**) and normalized absolute errors ${\bar{\Delta }}_{1,1}({\bar{t}}_{n})\times 10^{-2}$ (**b**) when the input test signal is $v(\bar{t})=-0.475\sin(\bar{t})$ .

**Figure 8:**
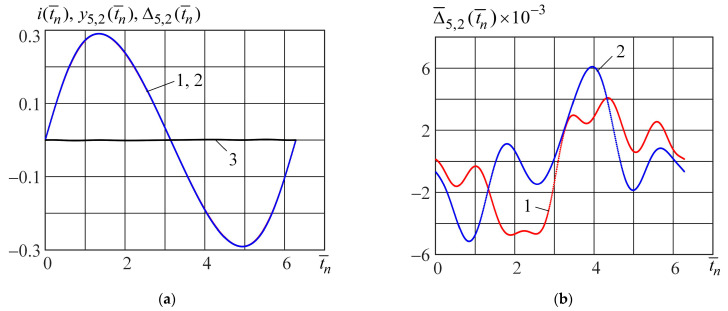
The output signals $i({\bar{t}}_{n}),\;y_{5,2}({\bar{t}}_{n}),\;\Delta_{5,2}({\bar{t}}_{n})$ (**a**) and normalized absolute errors ${\bar{\Delta }}_{5,2}({\bar{t}}_{n})\times 10^{-3}$ (**b**) when the input test signal is $v(\bar{t})=0.75\sin(\bar{t})$ .

Figure 7a shows the following curves: curve 1 (blue) is the output signal calculated from Equation (24) when the test signal is $v(\bar{t})=-0.475\sin(\bar{t})$ ; curve 2 (red) is the output signal $y_{k,\theta }({\bar{t}}_{n})$ ( k=1 and θ=1 ) of the piecewise neural model; and curve 3 (black) is the absolute error obtained from Equation (26). In Figure 7b, the normalized absolute errors (Equation (27)) of modeling using the piecewise neural (curve 1, red) and polynomial (curve 2, blue) models are represented. Figure 8a,b shows curves similar to Figure 7a,b when the test signal is $v(\bar{t})=0.75\sin(\bar{t})$ .

Analysis of Figure 6, Figure 7 and Figure 8 shows that the piecewise neural model ensures a higher modeling accuracy of the Bernoulli memristor on the set of harmonic signals (21) compared with the piecewise polynomial model. The complexities of these models are nearly equal. The parameter number of the piecewise neural model is 107, and the parameter number of the piecewise polynomial model is 100.

## 4. Conclusions

Due to the variety of physical processes underlying the technology and wide application of memristors (with different signals, operating modes, and performance requirements), the task of building of the universal behavioral models of memristors and memristor-based devices can be challenging. Behavioral models represent an object as a “black or gray box” and establish a mathematical correspondence between the sets of the input and output signals.

The universal behavioral model based on the split method is represented. According to this method, the operator of a device comprises two operators. The first operation is the splitting of the scalar input signal, i.e., converting it into the split vector signal. The second operation is the mapping (nonlinear, inertialess transformation) of the set of the split vector signals into the set of the output scalar signals. The splitting of the vector signals provides the uniqueness of the mentioned mapping. These two operations result in the behavioral model, which approximates the correspondence between the sets of the input and output signals.

The behavioral model based on split signals has the following advantages:

- It is universal and, therefore, invariant to physical processes and applicable to different signals and types of memristors and memristor-based devices;
- It is functional under strong nonlinearity; thus, it surpasses the Volterra series, which is limited by convergency;
- It is adaptive to the input signals because these signals are split. In a sense, the model loses its universality (excessive one) and, hence, its complexity. As a result, we are approaching a compromise between building a simple model and achieving high accuracy;
- It is necessary when the description of a device is insufficient or complex.

The splitter structure of harmonic signals is proposed. These signals are used to model different dynamic systems, including memristors and memristive devices. The minimum number of the harmonic signal splitting channels based on the delay line has been determined. This number establishes the minimum dimension of the nonlinear model. Thus, the behavioral model is simplified already at the stage of splitting the input signal.

To increase accuracy, the piecewise behavioral models are represented. A piecewise model is the combination of the models, which are built on the subsets of the input signals and whose complexities (the powers of multidimensional polynomials or the number of neurons) are lower than the complexity of a general model built on the entire assigned set of the input signals. Thus, the high-dimensional operator approximation is divided into several sub-approximations with lower dimensions. This approach solves the poor conditionality problem relevant to polynomials and overcomes the convergence difficulties relevant to training neural networks.

Piecewise models with split signals are used for the behavioral modeling of Bernoulli memristors, whose current dynamics are described using Bernoulli nonlinear differential equations. For the harmonic input signal, the split signal vector is formed using a delay line. The elements of the split vector form basic functions in piecewise polynomial and piecewise neural models. Comparative analysis has shown that the piecewise neural model ensures a higher accuracy than the piecewise polynomial, and the complexities of both models are almost the same (with nearly equal numbers of the model parameters).

The positive results obtained in the modeling of the Bernoulli memristors expand the application of signal splitting behavioral models. The above-mentioned advantages of the proposed models are a basis for modeling not only memristors with different physical processes but also memristive devices, which are synthesized on the basis of different technologies, have severely nonlinear characteristics, and deal with different signals.

## Data Availability

Data are contained within the article.
